# A mutation in *LacDWARF1* results in a GA-deficient dwarf phenotype in sponge gourd (*Luffa acutangula*)

**DOI:** 10.1007/s00122-021-03938-4

**Published:** 2021-08-14

**Authors:** Gangjun Zhao, Caixia Luo, Jianning Luo, Junxing Li, Hao Gong, Xiaoming Zheng, Xiaoxi Liu, Jinju Guo, Lingyan Zhou, Haibin Wu

**Affiliations:** 1 Guangdong Key Laboratory for New Technology Research of Vegetables, Vegetable Research Institute, Guangdong Academy of Agricultural Sciences, Guangzhou, 510640 Guangdong China; 2 College of Agriculture & Biology, Zhongkai University of Agriculture and Engineering, Guangzhou, 510225 Guangdong China

## Abstract

**Key message:**

A dwarfism gene *LacDWARF1* was mapped by combined BSA-Seq and comparative genomics analyses to a 65.4 kb physical genomic region on chromosome 05.

**Abstract:**

Dwarf architecture is one of the most important traits utilized in Cucurbitaceae breeding because it saves labor and increases the harvest index. To our knowledge, there has been no prior research about dwarfism in the sponge gourd. This study reports the first dwarf mutant WJ209 with a decrease in cell size and internodes. A genetic analysis revealed that the mutant phenotype was controlled by a single recessive gene, which is designated *Lacdwarf1* (*Lacd1*). Combined with bulked segregate analysis and next-generation sequencing, we quickly mapped a 65.4 kb region on chromosome 5 using F_2_ segregation population with InDel and SNP polymorphism markers. Gene annotation revealed that *Lac05g019500* encodes a gibberellin 3β-hydroxylase (GA3ox) that functions as the most likely candidate gene for *Lacd1*. DNA sequence analysis showed that there is an approximately 4 kb insertion in the first intron of *Lac05g019500* in WJ209. *Lac05g019500* is transcribed incorrectly in the dwarf mutant owing to the presence of the insertion. Moreover, the bioactive GAs decreased significantly in WJ209, and the dwarf phenotype could be restored by exogenous GA_3_ treatment, indicating that WJ209 is a GA-deficient mutant. All these results support the conclusion that *Lac05g019500* is the *Lacd1* gene. In addition, RNA-Seq revealed that many genes, including those related to plant hormones, cellular process, cell wall, membrane and response to stress, were significantly altered in WJ209 compared with the wild type. This study will aid in the use of molecular marker-assisted breeding in the dwarf sponge gourd.

**Supplementary Information:**

The online version contains supplementary material available at (10.1007/s00122-021-03938-4).

## Introduction

The sponge gourd (2*n *= 26) is an important vegetable and medicinal plant in tropical and subtropical regions, and has a long history of cultivation in Asian and African tropical countries (Kalloo 1993; Wu et al. 2014, 2016). The sponge gourd is a member of the Cucurbitaceae and has nine species, but only *Luffa acutangula* (L.) Roxb. and *L. cylindrica* (L.) Roem. are domesticated (Gautam et al. 2017; Wu et al. 2014). Its tender fruits are rich in vitamin A, vitamin C, and iron and have high nutritional value (Dubey et al. 2015; Xu et al. 2008). In addition, the sponge gourd possesses multiple potential biological and therapeutic activities in the management of hepatoprotective, antidiabetic, antiulcer, anticancer, fungistatic, analgesic, antimicrobial, immunomodulatory(Kuppast and Mankani 2012; Shendge and Belemkar 2018), and anti-HIV-1 activities(Ng et al. 2011).

Height is one of the most important traits in plant breeding. Dwarfism can save labor in management and harvesting, improve lodging resistance and increase the harvest index (Peng et al. 1999). The semi-dwarf variety of rice (Sasaki et al. 2002; Suh 1978), increases the yield potential and nitrogen responsiveness, resulting in an increase in the harvest index of more than 60% (Khush 2001; Peng et al. 1999), which constitutes an important breakthrough in the history of crop improvement. This remarkable achievement is referred to as the “Green Revolution” (Khush 2001; Peng et al. 1999).

Over recent years, dozens of genes regulating plant height have been identified from rice, wheat, cucumber, and other plants (Hou et al. 2017; Li et al. 2013; Magome et al. 2004; Sasaki et al. 2002). Many of these genes affect the synthesis and signal transduction of hormones, such as gibberellin(Sasaki et al. 2002; Ueguchi-Tanaka et al. 2005), brassinolide(Chory et al. 1991; Hou et al. 2017; Schwessinger et al. 2011), auxin (Li et al. 2018) and strigolactones (Kohlen et al. 2012) that affect cell elongation or development.

Gibberellins (GAs) regulate diverse plant developmental processes, such as seed germination, stem elongation, flowering, and fruit development (Silverstone and Sun 2000; Wang et al. 2015). Dwarfs will be formed if the genes in GA synthesis or signal transduction are mutated. For example, mutations in *ent-kaurenoic acid oxidase (KAO)* (Fambrini et al. 2011; Regnault et al. 2014), *ent-copalyl diphosphate synthase* (*CPS)* (Magome et al. 2004), the well-known “Green Revolution” genes *GA20-oxidases* (Sasaki et al. 2002; Zhai et al. 2019), or *GA3ox* (Chen et al. 2014; Mitchum et al. 2006; Wei et al. 2019) in GA synthesis, reduce the levels of endogenous GAs and lead to dwarfism. The GAs receptor (gibberellin-insensitive dwarf protein1, GID1) (Shimada et al. 2008) and several repressor proteins (DELLA) (Murase et al. 2008) form a complex to precisely regulate the responses of plants to GAs.

Bulked segregant analysis (BSA) coupled with next-generation sequencing (NGS) provides a rapid and efficient method to map genes and QTLs (Takagi et al. 2013). BSA was used to identify markers using bulked sample pools (Michelmore et al. 1991). Whole-genome sequencing can be a powerful tool for the identification of variations between different varieties (Bentley 2006). The improvement in technologies and substantial reduction in the cost of NGS enabled the coupling of whole-genome resequencing with BSA (BSA-Seq). BSA-Seq has been successfully used in many crops (Zou et al. 2016), including cucumber (Song et al. 2020), watermelon (Wei et al. 2019) and melon (Zhang et al. 2019).

Dwarfism is an important trait in Cucurbitaceae breeding. Dwarf plants are suitable for intercropping, and in turn improve the index of multiple cropping and increase the yield per unit area (Li et al. 2016). Owing to its contribution to yield and reduction in labor in management and harvesting, dwarfism is an important agronomic trait for selection in Cucurbitaceae breeding. Until now, there have been many studies on dwarf vines in many Cucurbitaceae crops including pumpkin (Zhang et al. 2015), cucumber (Hou et al. 2017), watermelon (Dong et al. 2018; Hexun et al. 1998; Wei et al. 2019) and melon (Hwang et al. 2014). In addition, a compact cucumber has been proposed for use in the high-wire cultivation of European greenhouse cucumbers (Li et al. 2011). Sponge gourd has similar growth habits with other cucurbitaceous crops, and short vines are also important breeding traits. However, to our knowledge, there have not been any relevant report studies conducted on it.

In this study, we identified a dwarf mutant of sponge gourd, WJ209, and genetic analysis showed that the mutant phenotype was controlled by a single recessive gene. By using BSA-Seq, we identified *Lac05g019500*, which encoded a gibberellin 3β-hydroxylase (GA3ox), the key enzyme in bioactive GAs production, and served as the most likely candidate gene for the dwarfism phenotype of WJ209. An insertion of approximately 4 kb was found in the first intron of mutant allele. This insertion caused the incorrect transcription of *Lac05g019500*, resulting reduction in the amount of bioactive GAs in WJ209, which is responsible for the dwarfism phenotype in sponge gourd. This study could aid in the use of molecular marker-assisted breeding in the dwarf sponge gourd.

## Materials and methods

### Plant materials and populations

The WT is an inbred line of sponge gourd. WJ209, the dwarf mutant, is a natural mutation obtained from the field production process of WT. After three generations of selfing, WJ209 was crossed to S1174 (the inbred line for whole-genome sequencing), and the F_1_ was self-pollinated to produce F_2_ progeny. The 323 S1174 × WJ209 F_2_ was used to examine the segregation and identification of candidate gene.

### Histological analysis of WT and WJ209

The third internode down from the growth point at 5-week-old plants was cut into 3-mm pieces and fixed in a formaldehyde-acetic acid-alcohol (FAA) solution (70% alcohol: 3.8% formalin: glacial acetic acid = 18:1:1) fixative for 24 h. The samples were then dehydrated with different concentrations of ethanol, embedded in paraffin, cut into 10-μm slices, stained with 1% toluidine blue for 5 min, and washed with deionized water and ethanol (Sun et al. 2017). Next, the paraffin sections were observed and imaged using LSM710 microscopy (Zeiss, Germany).

### WT and WJ209 treatment with exogenous GA_3_

The germinated seeds were sown in plastic pots (size: 7 × 7 × 7 cm) and grown at 26 ± 2 °C in a climate-controlled room with a 16 h/8 h light/dark cycle. At the two-leaf stage, the treated leaves were sprayed with 100 μM GA_3_, while the controls seedlings were sprayed with water. Three biological replicates, each with 10 plants, were sprayed four times once every other day. The height from cotyledons to the growing point was measured 3 days after the final treatment. In addition, untreated WT and WJ209 seedlings at the three-leaf stage and whole plant were sampled to quantify the endogenous GAs and proceed with RNA sequencing. Three biological replicates, each with nine plants, were frozen in liquid nitrogen and stored at −80 °C until use.

### Quantify chlorophyll content

WT and WJ209 seedlings at the 3-week-old were used to quantify the chlorophyll content (SPAD) by using plant nutrient analyzer (TYS-4N, TOP Instrument, China). After instrument calibration, the first expanded leaf was clamped by instrument receiving window (ensure fully covered) and recorded the reading.

### Quantification of Endogenous GAs

The sample were prepared and analyzed as previously described (Plackett et al. 2012). In brief, fine powder sponge gourd seedlings (1 g) were extracted with 10 ml acetonitrile for 12 hrs at 4 °C. Deuterated gibberellin (OIChem, Czechoslovakia) was added to the plant samples to serve as an internal standard. After 5 mins of centrifugation at 12,000 g at 4 °C, the supernatant was removed. A solution of five times the volume of acetonitrile was added to the precipitate and extracted twice. The resulting supernatants were combined, and C18 and GCB were added to purify the impurities, and centrifuged at 12,000 g for 5 mins at 4 °C. The supernatant was then evaporated with nitrogen and reconstituted with 200 μl of methanol. The content of GAs was determined with an HPLC (AGLIENT1290, AGLIENT, USA) coupled with tandem mass spectrometry (SCIEX-6500Qtrap, AB SCIEX, USA).

### Whole-genome re-sequencing

Fifty normal height plants and 50 dwarf plants were randomly selected from the F_2_ population. Genomic DNA was extracted from each individual using the CATB method (Murray and Thompson 1980), and the samples were mixed into two DNA pools: one of N-bulk for normal height plants and one for M-bulk for dwarfs. Simultaneously, the WJ209 genomic DNA was extracted to serve as the parent pool. Sequencing libraries from each pool were generated using a Truseq Nano DNA HT Sample Preparation Kit (Illumina, USA) following the manufacturer’s recommendations, sequenced by an Illumina HiSeq4000 platform, and 150-bp paired-end reads were generated. The depth of sequencing data of N-bulk and M-bulk was not less than 50×, and that of the parent pool were not less than 10×.

### Data analysis and marker development

The quality of the BSA-Seq data was evaluated using FastQC software (Andrews 2010). Clean reads were obtained by removing reads containing adapter or low-quality reads. BWA software (Li and Durbin 2009) was used to align the clean reads with the reference genome S1174 (data not shown) and convert Sam files to Bam files (Li et al. 2009). Potential PCR duplications were removed using SAMtools software (Li et al. 2009). GATK software (McKenna et al. 2010) was used to call variants between the N-bulk and M-bulk and between the parent-pool and reference genome, respectively. After removing the SNP/InDel index in both pools with less than 0.3 or more than 0.7, we calculated the delta SNP/InDel index using Perl scripts and drew a Manhattan Plot using R scripts. Primers of potential InDel-based markers and SNP markers between WJ209 and S1174 were designed using Primer Premier 5.0.

### RNA-Sequencing and data analysis

Sequencing libraries were generated using an NEBNext UltraTM RNA Library Prep Kit for Illumina (NEB) following the manufacturer’s instructions. The libraries were sequenced on an Illumina Nova-Seq platform, and 150-bp paired-end reads were generated. The quality of RNA-Seq data was evaluated using FastQC software (Andrews 2010). Clean data were obtained by removing adapter or low-quality reads from the raw data. The Hisat2 program (Kim et al. 2015) was used to align the clean reads with reference genome S1174 (data not shown), and the Sam files were converted to Bam files using SAMtools software (Li et al. 2009). The level of gene expression was quantified using featureCounts (Liao et al. 2014). The DESeq2 (Love et al. 2014) R package was used to determine the DEGs. Genes with an adjusted P value (padj) <0.05 and |log (fold change) |≥1 were assigned as differentially expressed.

### Amplification of the insertion

KOD-Plus-Neo (TOYOBO, code No. KOD-401) was used to amplify the insertion. PCR components included the following: 32 μl ddH_2_O, 5 μl 10 × PCR Buffer, 5 μl 2 mM dNTPs, 3 μl 25 mM·MgSO_4_, 1.5 μl 10 pmol/μl primer F, 1.5 μl 10 pmol/μl primer R, 1 μl KOD-Plus-Neo (1.0 U/μl), and 1 μl genomic DNA (100 ng/μl), in a total reaction volume of 50 μl. The PCR cycle conditions were as follows: pre-denaturation 94 °C 2 min, denaturation 98 °C 10 sec, annealing 58 °C 30 sec, extension 68 °C 2.5 min, 33 cycles, and 68 °C 5 min, with storage at 4 °C.

### GA3ox homolog identification and phylogenetic analysis

For the genome-wide identification of *GA3oxs* in other Cucurbitaceae species, including *Cucumis sativus*, *Cucumis melo*, *Citrullus lanatus*, *Cucurbita maxima* and *Lagenaria siceraria*, the genome and amino acid sequences were downloaded from the Cucurbit Genomics Database (http://cucurbitgenomics.org/), and amino acid sequences of *Arabidopsis thaliana GA3oxs* (*AtGA3OX1: AT1G15550.1*, *AtGA3OX2: AT1G80340.1*, *AtGA3OX3: AT4G21690.1*, *AtGA3OX4: AT1G80330.1*) were used as queries to blast against the protein file of other Cucurbitaceae species using the BLASTp program (*E-value *< 1e^−5^).

The amino acid sequences of GA3oxs were aligned using ClustalX (Larkin et al. 2007). MEGA7 (Kumar et al. 2016) was applied to construct the phylogenetic tree using the neighbor-joining method with 1000 bootstrap replicates. The motif of proteins was predicted using MEME online software (http://meme-suite.org/tools/meme) with the maximum number of motifs equal to 10 and the maximum width equal to 100.

## Results

### Morphology of the dwarf mutant

**Fig. 1:**
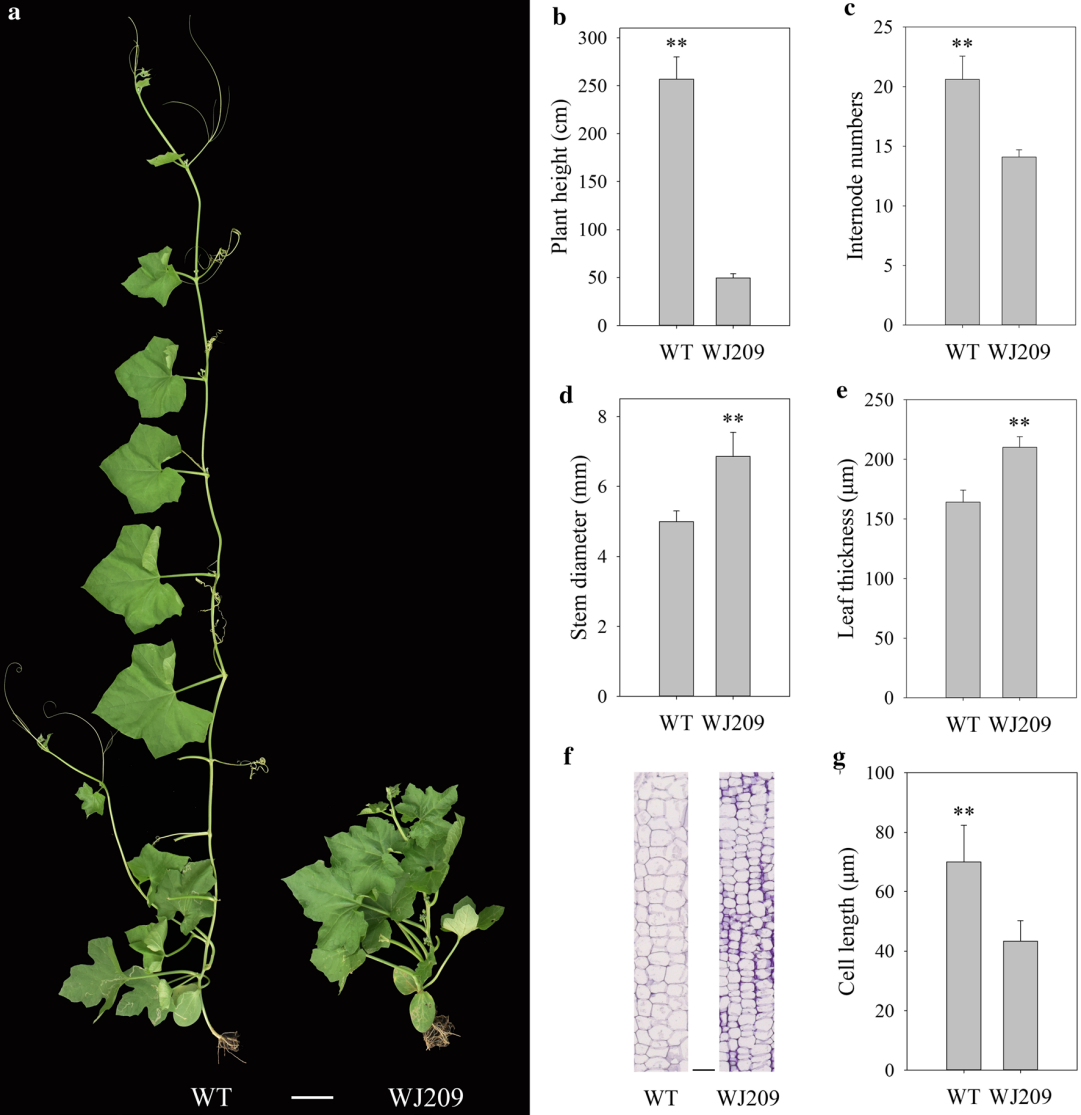
Phenotypic characterization of the normal line WT and dwarf mutant WJ209 of the sponge gourd. **a** Phenotypes of WT and WJ209. Bar, 10 cm. **b** Plant height. **c** Internode numbers. **d** Stem diameter. **e** Leaf thickness. **f** Longitudinal sections of the internodes. Bar, 100 μm. **g** The cell length is shown in f. The asterisks indicate significant differences (Student’s *t* test): **p < 0.01, *n* ≥ 15. Error bars represent the mean ± SD

The dwarf mutant WJ209 was much shorter than the wild type (WT) (Fig. 1a). The total length of WJ209 (49.4 ± 4.7 cm, 14 internodes) was reduced to 19.2% relative to the WT (256.8 ± 23.1 cm, 20 internodes) (Fig. 1b and c), while the stem diameter and leaf thickness increased (Fig. 1d and e). In addition, the leaf of WJ209 turned dark-green and chlorophyll content (SPAD) increased (Supplementary Fig. S1). Moreover, the cell length of longitudinal section significantly decreased in WJ209 (43.3 ± 6.9 μm) compared with that of the WT (70.1 ± 12.3 μm) (Fig. 1f, g).

### WJ209 is a GA biosynthetic-deficient mutant

**Fig. 2:**
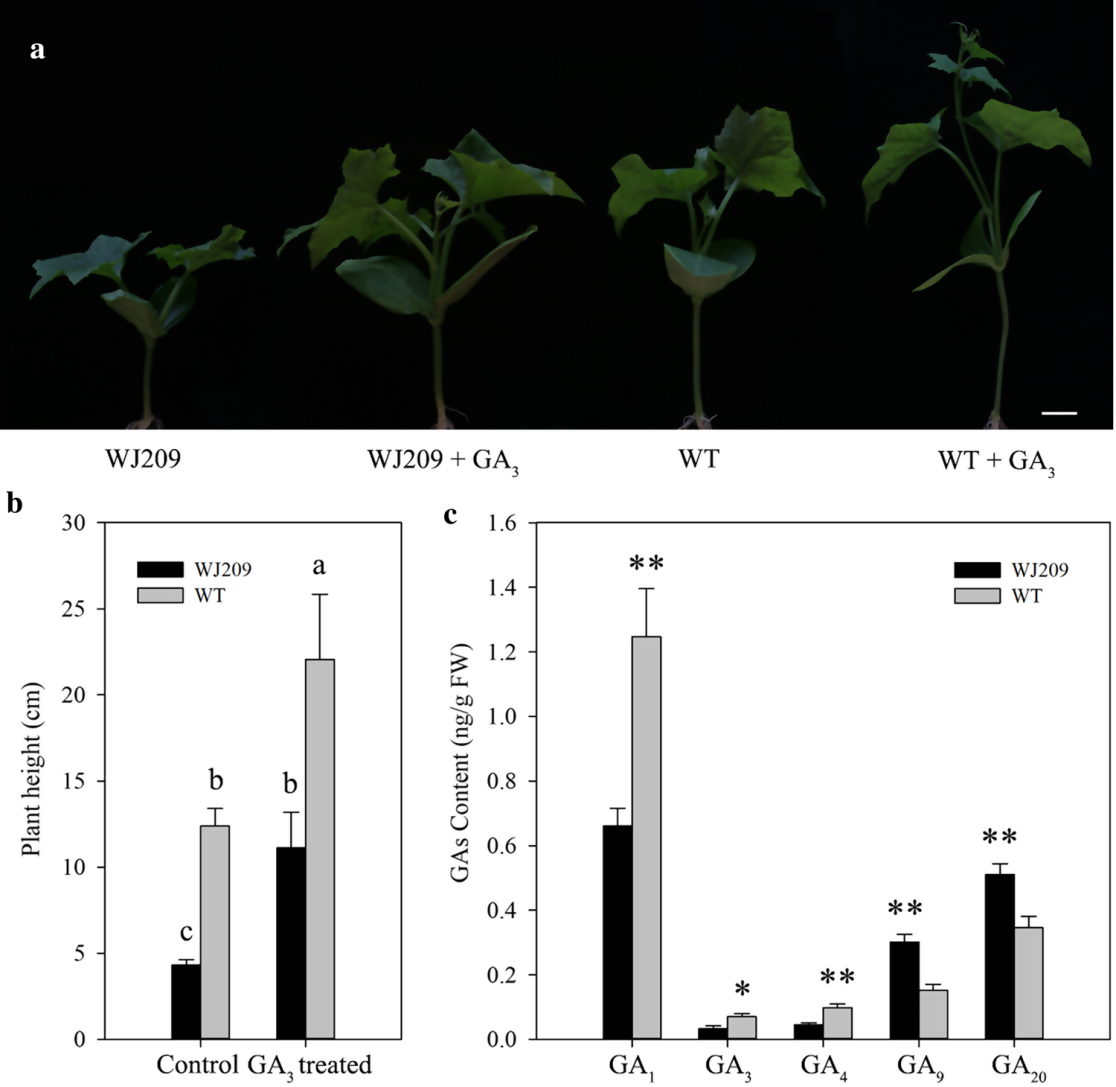
Recovery of the dwarf mutant by treatment with exogenous GA_3_. **a** Seedlings treated with 100 μm GA_3_ or water. Bar, 2 cm. **b** Height of the treated Seedlings. **c** Endogenous levels of GAs in WJ209 and WT. Different letters in b refer to their significance at *p* < 0.05 (Duncan’s test). The asterisks indicate significant differences (Student’s t test): *p < 0.05, **p < 0.01. Error bars represent the mean ± SD

Bioactive GAs play an essential role in stem elongation, and the plant will be dwarf if the genes involved in GAs syntheses are mutated (Sasaki et al. 2002; Silverstone and Sun 2000). To test whether GAs regulate the height of dwarf mutant, the mutant was treated with 100 μM GA_3_. GA_3_ promotes stem internode elongation, and the height of WJ209 and the WT increased significantly after treatment with exogenous GA_3_ (Fig. 2). In addition, the height of WJ209 (11.5 ± 2.4 cm) after treatment with exogenous GA_3_ did not differ from that of the WT (12.4 ± 1.0 cm) when it was treated with water (Fig. 2b). To confirm whether the biosynthesis of GA was impaired in WJ209, the levels of endogenous GAs were measured in WJ209 and the WT (Fig. 2c). The levels of bioactive GA_1_, GA_3_ and GA_4_ decreased significantly in WJ209 compared with the levels in WT, while the content of their immediate precursors, GA_9_ and GA_20_, increased (Fig. 2c), suggesting that the dwarf phenotype is associated with decreased levels of bioactive GAs. Taken together, these results confirmed that WJ209 is a GA biosynthetic-deficient mutant.

### Genetic analysis of the dwarf mutant

To analyze the genetic characteristics of the dwarf phenotype, WJ209 was crossed with S1174 (the inbred line for whole-genome sequencing). The F_1_ plants exhibited wild-type phenotypes, suggesting that the mutant trait is recessive. The F_2_ progeny exhibited a segregation ratio of 3:1 (Normal: Dwarfism = 235:88, *χ*^2 ^= 0.87; *P* = 0.352). Therefore, we concluded that the dwarf phenotype of WJ209 is controlled by a single recessive gene and designated it *Lacdwarf1* (*Lacd1*).

### Mapping of the *Lacd1* gene by BSA-Seq

**Fig. 3:**
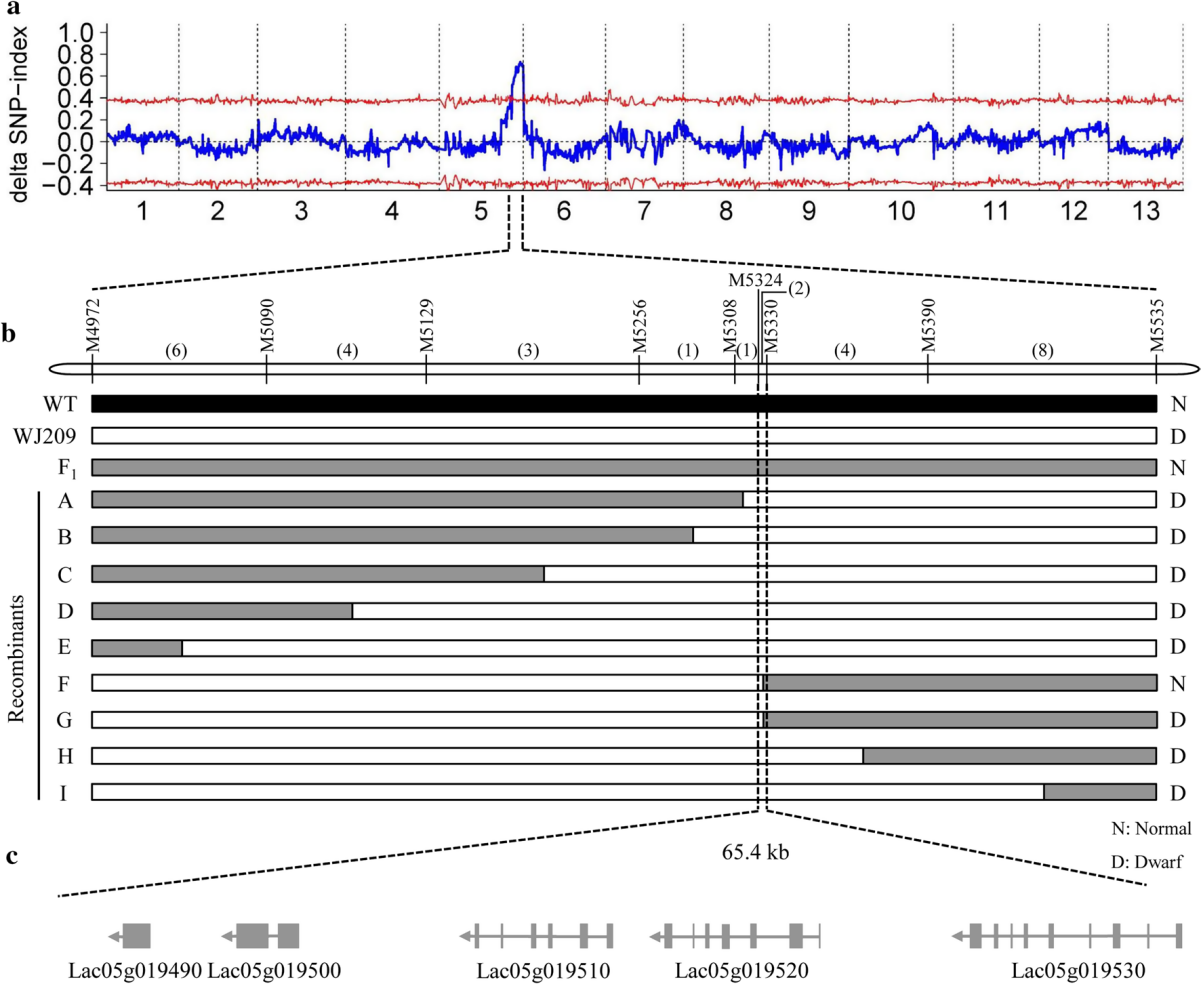
Mapping of the dwarfism gene. **a** Delta SNP-index distribution on chromosomes. **b** Fine mapping of *Lacd1.*The numbers within brackets indicate the number of recombinants. **c** Structure of the predicted genes in mapping interval

A bulked segregant analysis combined whole-genome re-sequencing (BSA-Seq) strategy was applied to rapidly identify *Lacd1* using 323 F_2_ plants from S1174 × WJ209. The dwarf mutant (WJ209), normal-type bulk (N-bulk) and mutant bulk (M-bulk) were obtained with 9.7 Gb (12.4×), 46.1 Gb (59.1×), and 45.8 Gb (58.7×) raw data, respectively (Supplementary Table S1). The Δ (SNP-index) was calculated and plotted against the genomic positions. The results revealed that there was only one candidate interval responsible for dwarfism in the 49.7−55.3 Mb region on chromosome 5 (Fig. 3a).

To fine-map *Lacd1*, we searched variants between WJ209 and S1174 in this candidate chromosome region, and then developed InDel and SNP markers (Supplementary Table S2). Polymorphic InDel and SNP markers were used to genotype the dwarf individuals of F_2_ population. First, a marker designated M5256 was designed at the peak position to detect the F_2_ population. We found 73 dominant homozygous lines, 165 heterozygous lines, and 85 recessive homozygous lines, and the ratio was consistent with Mendel’s law of segregation (*P *> 0.05). This indicates that there is a gene that controls vine growth near the peak. Molecular markers were developed in the target range to detect individual recombinant plants. The A–E individual plants had short vines, and the target gene was excluded from the region between M4972 and M5308 markers. The G–I individual plants also had short vines, and thus, the target gene was excluded from the region between M5330 and M5535. Simultaneously, the F individual plant displayed a normal vine phenotype, and therefore, the target gene was excluded from the region between M4972 and M5324 markers. A comprehensive analysis shows that the target gene is located between M5324 and M5330. Finally, *Lacd1* was fine-mapped in a 65.4 kb interval between the SNP markers M5324 and M5330 (Fig. 3b).

### Candidate gene analysis of *Lacd1*

**Fig. 4:**
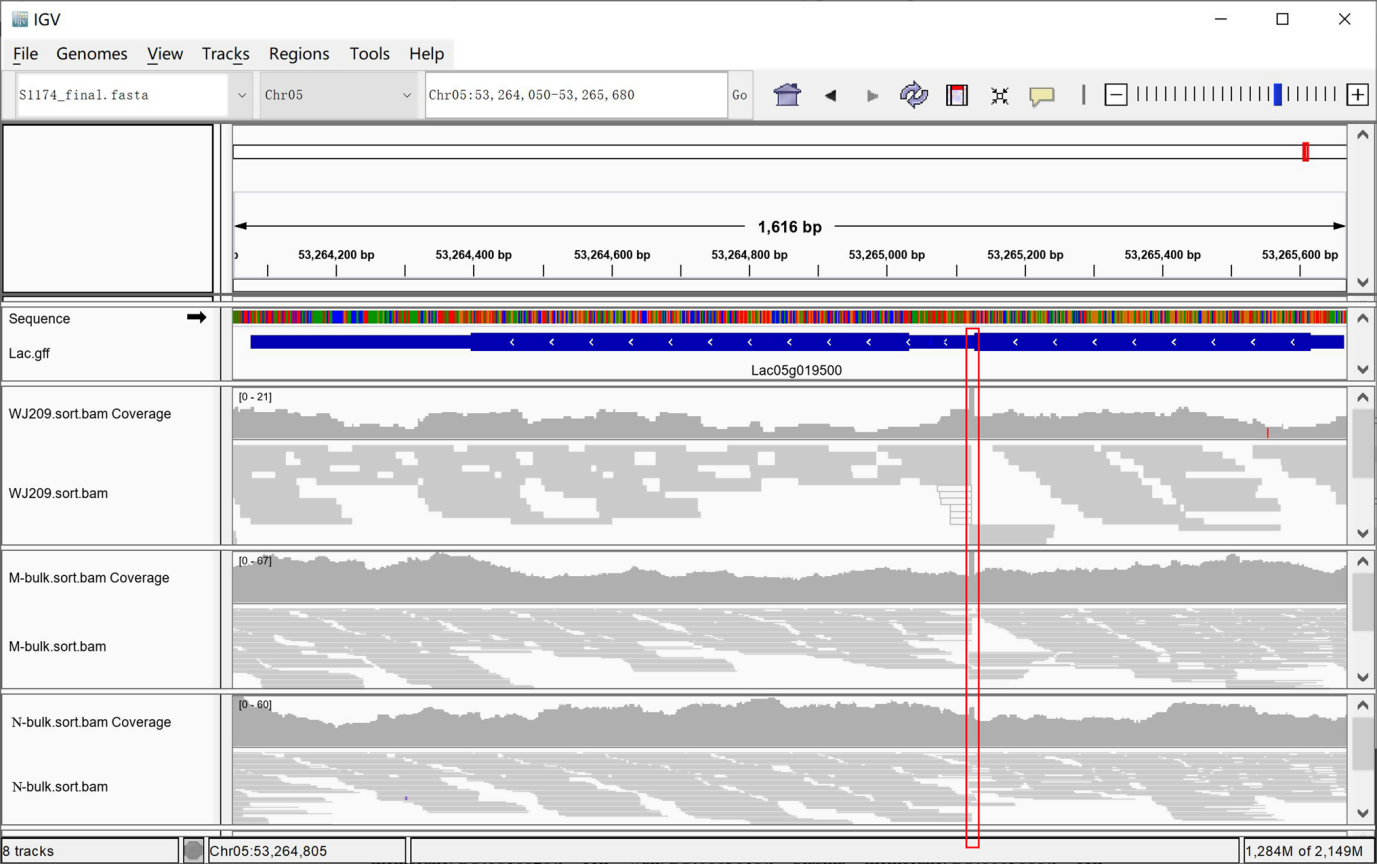
Mapped reads on *Lac05g019500*. The red rectangle shows potential insertions that were visualized by an Integrative Genomics Viewer (IGV) (Robinson et al. 2011) (colour figure online)

The genomic annotation indicated that the 65.4 kb interval encodes five genes (Fig. 3c, Table 1). There was no difference in gene expression and structure between WT and WJ209 in *Lac05g019490*, *Lac05g019510*, *Lac05g019520* and *Lac05g019530* based on the resultes of RNA-Seq (Supplementary Fig. S2) and Sanger sequencing after having amplified the gene in the target region. *Lac05g019520* encoded katanin p60 ATPase-containing subunit A1. Research shows that katanin p60 ATPase is involved in cellular process (Aragão et al. 2017). It's the same as *Lac05g019490, Lac05g019510,* and *Lac05g019530*, no studies have shown that the proteins encoded by *Lac05g019490, Lac05g019510,* and *Lac05g019530* were related to plant growth and development, and there was no difference in gene structure and expression between WT and WJ209. However, analyses of the sequencing data of WJ209 showed that there could be a large fragment insertion at the first intron of *Lac05g019500* (Fig. 4). The M-bulk also appeared to have this insertion; however, the N-bulk lacked it (Fig. 4). Simultaneously, only the *Lac05g019500* expression decreased significantly in WJ209 compared with that of the WT (Supplementary Table S3). Next, a pair of primers (SV-F/R) was designed to amplify this potential insertion (Supplementary Table S2). As expected, a 243-bp DNA fragment was amplified by the primers in the WT, while it was approximately 4 kb in WJ209 (Supplementary Fig. S3). To confirm the genomic variations observed above, we cloned the insertion sequence in 323 F_2_ plants derived from S1174 × WJ209. All the dwarf plants were able to amplify the homozygous 4 kb insertion fragment, while the normal plants were able to amplify homozygous 243 bp DNA fragments or heterozygous fragments (243 bp and 4 kb insertion fragment). The RNA-Seq data indicated that this insertion resulted in the incorrect transcription of *Lac05g019500* and could not amplify the complete cDNA in WJ209 (Supplementary Fig. S4). It is notable that *Lac05g019500* encoded the gibberellin synthetic enzyme GA3-oxidase 1 (GA3ox1), which is the key enzyme in the production of bioactive GAs. Taken together with the fact that WJ209 is a GA biosynthetic-deficient mutant, the dwarf phenotype of WJ209 was probably caused by the mutation in *Lac05g019500*. Therefore, *Lac05g019500* is the *Lacd1* gene.

**Table 1:** Genes in the mapping interval

Gene ID	Annotation
*Lac05g019490*	LINE-1 retrotransposable element ORF2 protein
*Lac05g019500*	Gibberellin 3-beta-dioxygenase 1-like
*Lac05g019510*	Uncharacterized
*Lac05g019520*	Katanin p60 ATPase-containing subunit A1
*Lac05g019530*	Aromatic aminotransferase ISS1

### Differentially expressed genes in WJ209 and WT

**Fig. 5:**
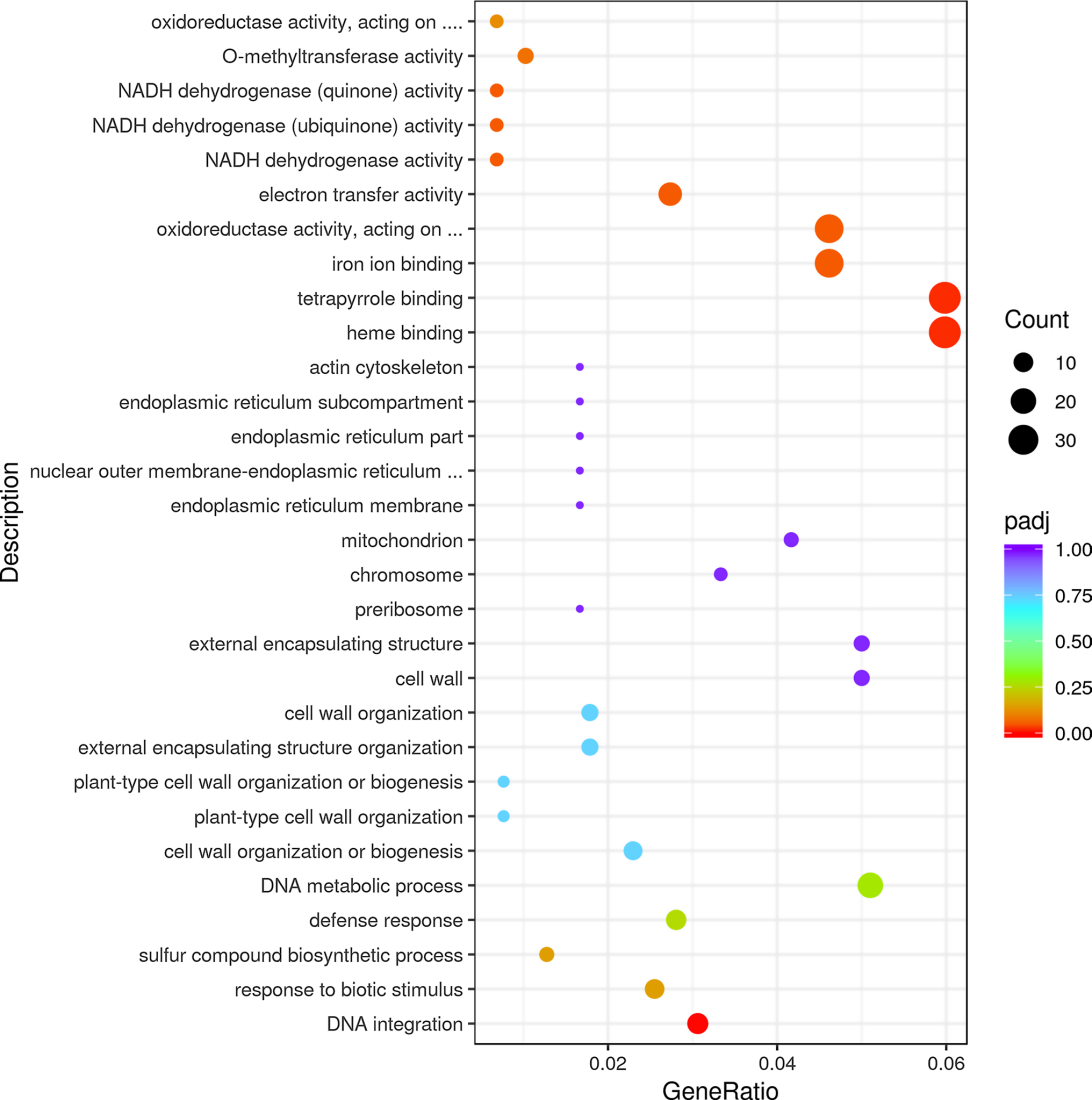
GO terms of the DEGs in WJ209 compared to WT

To investigate the molecular mechanisms that underlie the WJ209 dwarf, a comparative transcriptome analysis was used to identify the differentially expressed genes (DEGs) between WJ209 and WT. There were 894 DEGs between the WJ209 and WT, including 408 up-regulated and 486 down-regulated genes. Gene Ontology enrichment revealed that many significantly changed genes were enriched in cellular processes (Fig. 5, Supplementary Tables S4 and S5), such as cellular process (GO:0009987) and cellular anatomical entity (GO:0110165). It is particularly notable that the expression of many genes related to cellular metabolic process (GO:0044237), membrane (GO:0016020), cell wall (GO:0005618), plasma membrane (GO:0005886), cell periphery (GO:0071944) and intrinsic component of membrane (GO:0031224), was significantly reduced in WJ209 (Fig. 5, Supplementary Fig. S5 and Supplementary Table S4). We also found that plant hormone metabolic, transport, response and signaling pathway-related genes were significantly changed, including the hormone biosynthetic process (GO:0042446), regulation of hormone levels (GO:0010817), hormone metabolic process (GO:0042445), response to hormone (GO:0009725), cellular response to hormone stimulus (GO:0032870), hormone transport (GO:0009914) and hormone-mediated signaling pathway (GO:0009755) (Supplementary Fig. S6, Supplementary Tables S4 and S5). Simultaneously, many significantly changed genes were enriched in responses to stress, such as response to stress (GO:0006950), response to stimulus (GO:0050896), defense response (GO:0006952), and response to biotic stimulus (GO:0009607) (Fig. 5, Supplementary Tables S4 and S5).

### Analysis of the expression of GAs biosynthetic and signaling pathways and regulatory genes

**Fig. 6:**
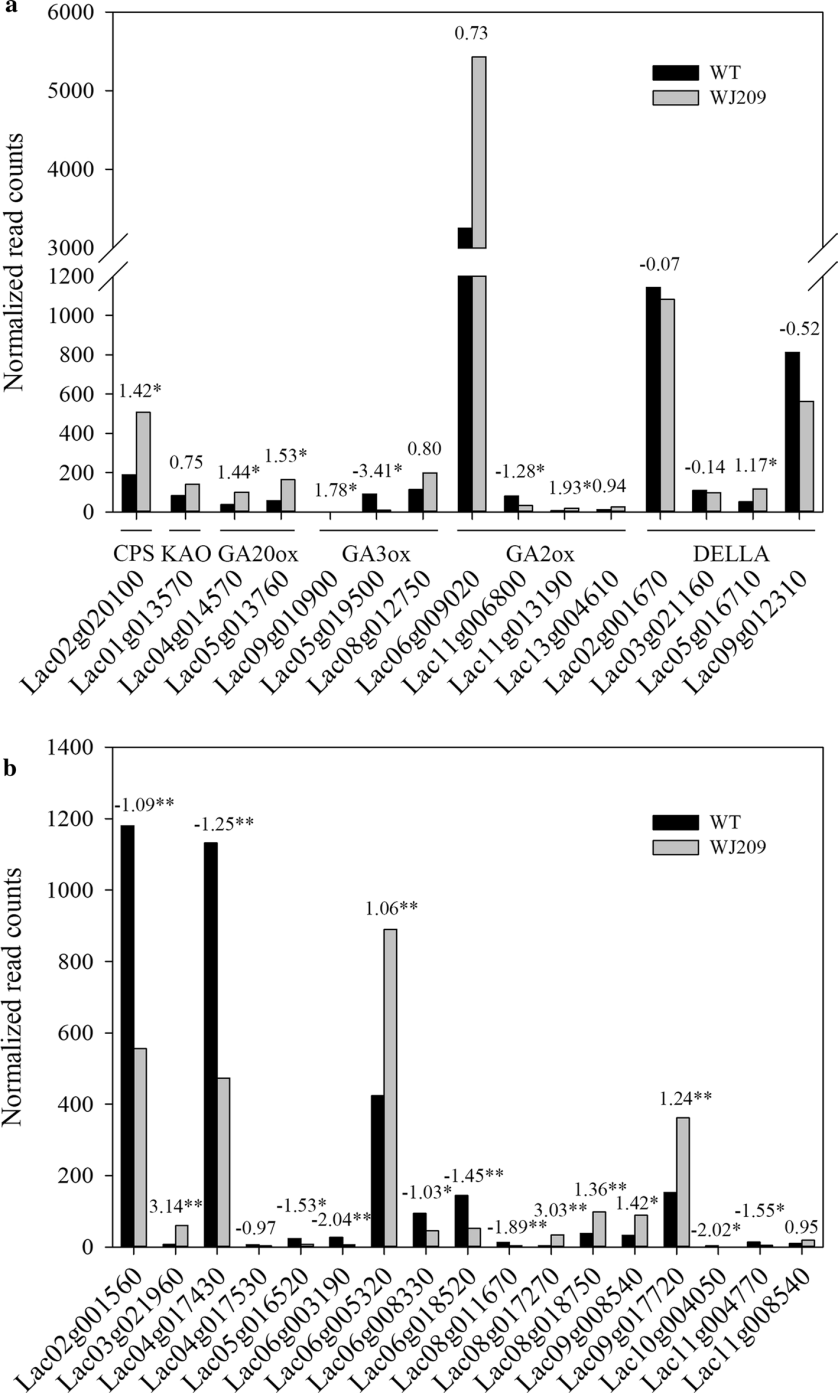
Analysis of the expression of GA biosynthetic and signaling pathways (**a**) and genes regulated by GAs (**b**). The numbers above the column are log2 [Foldchange (WJ209/WT)]. The asterisks indicate significant differences (Student’s *t* test): *padj < 0.05, **padj < 0.01

The synthesis of bioactive gibberellin is a complex process, and the genes that encode functional enzymes at each step have been well characterized (Claeys et al. 2014; Hedden and Proebsting 1999). To study the pattern of expression of the GAs biosynthetic pathway-related genes in WJ209, we utilized the homologous amino acid sequences from Arabidopsis as queries and combined them with genomic annotation. We retrieved one *CPS*, one *KAO*, three *GA20oxs*, two *GA3oxs*, four *GA2oxs*, and four *DELLAs* (Fig. 6a). In addition, we retrieved 17 genes regulated by GAs based on genomic annotation and GO annotation (Fig. 6b). Compared with the profiles of expression of the WT, the expression of *Lacd1* gene (*Lac05g019500*) decreased significantly in WJ209 (Fig. 6a). The other GAs biosynthetic-related genes, including *CPS*, *KAO*, *GA20ox*, and an additional *GA3ox*, were up-regulated (Fig. 6a). Similarly, three *GA2ox* homologs were up-regulated in WJ209, while only one (*Lac11g006800*) was significantly down-regulated (Fig. 6a). In addition, one *DELLA (Lac05g016710)* was significantly up-regulated, while three other *DELLAs* had no obvious difference in WJ209 compared with that of the WT (Fig. 6a). In addition, nine genes regulated by GAs were significantly down-regulated, while eight genes regulated by GAs had no obvious difference or were up-regulated in WJ209 compared with that of the WT (Fig. 6b).

### Phylogenetic analysis of *GA3oxs* in the Cucurbitaceae

**Fig. 7:**
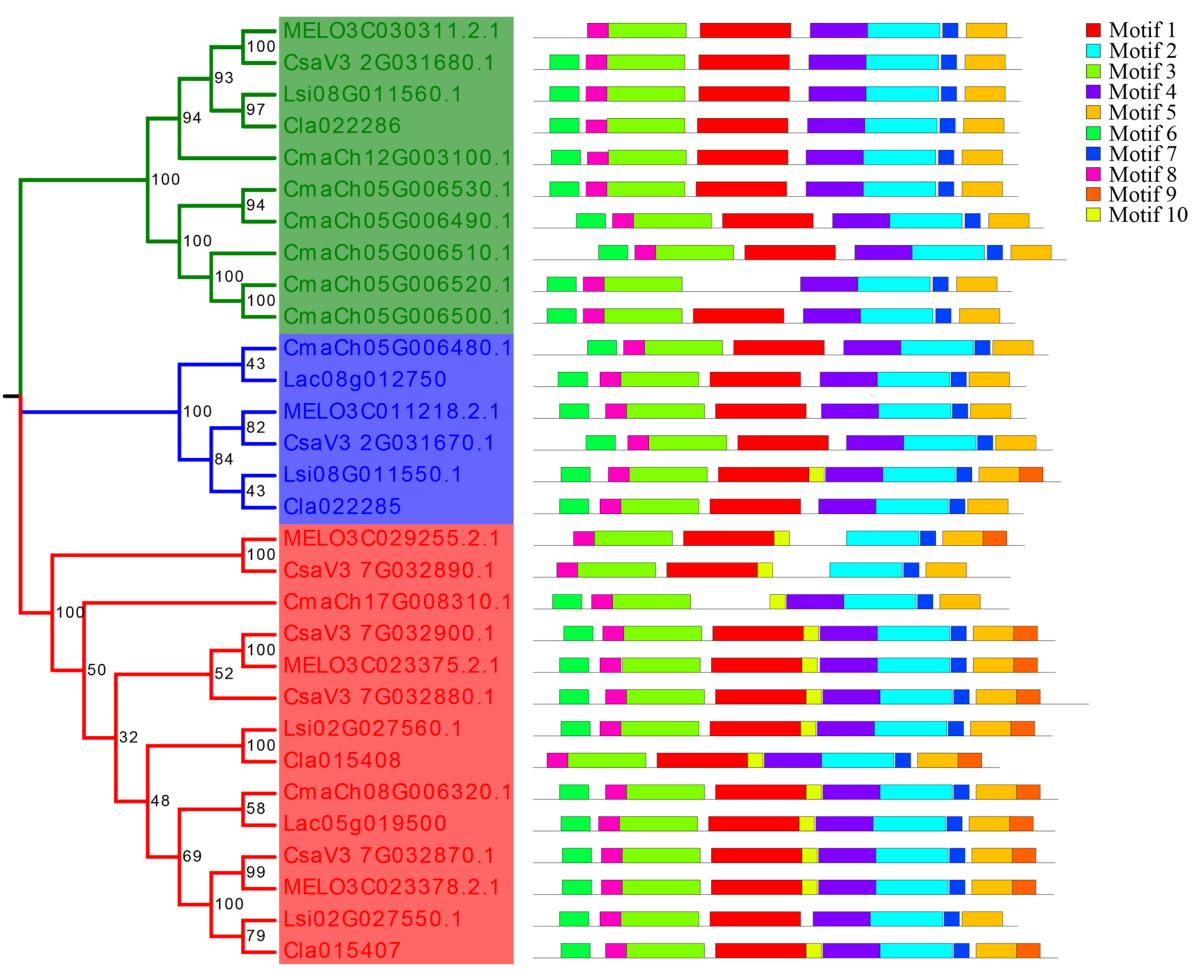
Phylogenetic and motif analyses of the *GA3ox* gene family in the sponge gourd and other species of Cucurbitaceae

To better understand the relationship between *Lacd1* and its homologues in other species in the Cucurbitaceae family, we identified 28 homologs from other cucurbit species, including *Cucumis sativus*, *Cucumis melo*, *Citrullus lanatus*, *Cucurbita maxima* and *Lagenaria siceraria* (Fig. 7). The results of the neighbor-joining tree indicated that the *GA3oxs* genes were divided into three groups with similar motifs shown by conservative motif analysis, and many of the amino acids in the motif were highly conserved (Fig. 7, Supplementary Fig. S7).

## Discussion

The sponge gourd is an important vegetable and medicinal plant in tropical and subtropical regions. Plant height is one of the most important traits in Cucurbitaceae breeding. Dwarfism can save labor in management and harvesting, improve lodging resistance and increase the harvest index (Peng et al. 1999). To our knowledge, no prior research about dwarfism in the sponge gourd has been conducted. In this study, we first identified a GAs biosynthetic-deficient dwarf mutant WJ209 in which the cell size and internode numbers decreased (Fig. 1). A genetic analysis revealed that the phenotype is controlled by a single recessive gene. We quickly located and cloned the target gene *Lacd1* using BSA-Seq and found that the gene is essential for bioactive gibberellin biosynthesis. In combination with RNA-Seq, we found that many genes, including the response to plant hormones, cellular process, cell wall, membrane and response to stress, were significantly altered in the dwarf mutant WJ209. This study could aid in the use of molecular marker-assisted breeding in dwarf sponge gourd.

There are various reasons that a plant might exhibit a dwarf phenotype, but GAs are one of the most important (Sasaki et al. 2002). Studies have shown that GA3ox catalyzes GA_9_ and GA_20_ without biological activity toward bioactive GA_1_ and GA_4_ (Hedden and Proebsting 1999). The bioactive synthesis of GAs is blocked in the *GA3ox* mutant, which affects the elongation of plant cells and causes plant dwarfing (Ágnes et al. 2008; Chen et al. 2014; Mitchum et al. 2006). In this study, the level of GA_1_, GA_3_ and GA_4_ decreased significantly compared with that in WT (Fig. 2c). This showed that WJ209 is a GAs biosynthetic-deficient mutant. A combination of DNA and RNA sequencing showed that the large fragment insertion of *Lacd1 (LacGA3ox1)* in WJ209 prevented it from correctly encoding the amino acid, resulting in the loss of the function of GA3 oxidase and reduced contents of bioactive GAs, which is responsible for the dwarfism phenotype in sponge gourd line WJ209.

It is a complex process to synthesize bioactive gibberellin (Claeys et al. 2014; Hedden and Proebsting 1999). In this study, the expression of *CPS, KO*, *GA20oxs* and another *GA3ox* homolog increased in WJ209 (Fig. 6), and the levels of GA_9_ and GA_20_ increased, while the bioactive GA_1_, GA_3_ and GA_4_ decreased (Fig. 2c). The same phenomenon was observed in previous work (Regnault et al. 2014; Shao et al. 2020; Wei et al. 2019), thus suggesting that there may be feedback regulation in GA biosynthesis. It is essential for plants to be able to precisely regulate their content of GAs and possess the ability to rapidly change this parameter in response to changes in their environment (Hedden and Thomas 2012). GA2oxs transforms bioactive gibberellin into inactive gibberellin and convert GA_9_ and GA_20_, which are substrates of GA3oxs, to GA_29_ and GA_51_ to regulate homoeostasis and allow a rapid reduction of the concentration of bioactive GAs when required (Aragão et al. 2017). In this study, the expression of three *GA2oxs* was up-regulated in WJ209 (Fig. 6). However, one *GA2ox* gene exhibited a distinct pattern of expression (Fig. 6), suggesting that this gene could perform distinct functions. Gibberellins regulate gene expression by promoting the degradation of the transcriptional regulator DELLA proteins (Murase et al. 2008). In our study, the expression of one *DELLA* gene increased, while the others did not change significantly (Fig. 6). Previous studies have shown that the expression of *DELLA* increased in GA-deficient mutants (Wei et al. 2019), while some exhibited no changes (Wang et al. 2016; Wei et al. 2019). DELLA played distinct but also overlapping functions in the repression of responses to GAs (Davière and Achard 2013).

A substantial amount of research indicates that plant hormones interact with each other to regulate plant growth and development (Blázquez et al. 2020; Depuydt and Hardtke 2011; Liu et al. 2018; Weiss and Ori 2007). Auxin promotes cell elongation partly by promoting GA3oxs and GA20oxs, inhibiting GA2oxs to increase the synthesis of GAs, and promoting the degradation of DELLAs which serve as negative regulators of the GAs signaling pathway, and then enhances GAs signaling pathway(Fu and Harberd 2003; Weiss and Ori 2007). Brassinosteroids (BRs) and GAs promote many similar developmental responses in plants, and BRs and GAs crosstalk through a direct interaction between GA-inactivated DELLA and BR-activated BZR1 (Bai et al. 2012; Nolan et al. 2020). Abscisic acid (ABA) and GAs control seed germination and the establishment of photoautotrophy by their antagonistic activity (Chen et al. 2020; Liu et al. 2019; Miao et al. 2019). In this study, a number of genes related to the hormone signaling pathway were significantly changed in WJ209 (Supplementary Fig. S6). For example, genes involved in the auxin and ABA signaling pathway were up-regulated, while those in the BR signaling pathway were down-regulated. These results showed that the hormones interacted and then regulated the expression of genes related to the cell wall, membrane and cellular process.

In recent years, an increasing amount of evidence shows that gibberellins are involved in the plant response to abiotic stress, including cold, salt and osmotic stress (Achard et al. 2006; Colebrook et al. 2014; Wang et al. 2020). Reducing the level of bioactive GAs in a GA-deficient biosynthetic mutant and blocking GAs signal transduction in a quadruple-*della* mutant enhanced the survival rate when *Arabidopsis thaliana* was subjected to salt and cold stress (Achard et al. 2006; Achard et al. 2008). In this study, many of the genes enriched in GO terms, including the responses to stress, stimuli and defense, were significantly altered in the dwarf mutant WJ209 (Fig. 5, Supplementary Tables S4 and S5). This finding improves our understanding of the potential mechanisms by which GAs modulate stress tolerance.

ATP-dependent caseinolytic proteases (Clp) in higher plants are important for chloroplast function and plant development (Mamaeva et al. 2020; Nishimura et al. 2015). Knockdown or mutant *clp* can affect the synthesis of chloroplast and eventually lead to yellow leaf in Arabidopsis (Clarke et al. 2005; Nishimura et al. 2015) and tobacco (Moreno et al. 2017). The growth of GA-deficient mutants of Arabidopsis (Dayan et al. 2010), rice (Sakamoto et al. 2004) and maize (Chen et al. 2014) resulted in production of dark-green leaves. We also found that the leaves of WJ209 turned dark-green (Supplementary Fig. S1). GO terms analyses showed that many of the up-regulated genes were enriched in the chloroplastic endopeptidase Clp complex (GO:0009840) and chloroplast (GO:0009507) (Supplementary Table S5). These probably increase the the content of chloroplast in WJ209, then increased chlorophyll content. This finding suggests that GA may affect chloroplast formation by regulating these genes.

## Conclusions

Using a BSA-Seq strategy, we identified a dwarf mutant gene, designated *Lacd1*, from a sponge gourd dwarf mutant (WJ209). Our results show that *Lacd1* encodes a GA3ox, which is one of the key enzymes in the GAs biosynthetic pathway. Sequencing analysis showed that there was an approximately 4 kb insertion in the first intron of the *Lacd1* gene in WJ209. This insertion caused incorrect transcription of the *Lacd1* gene, resulting in the reduced amount of bioactive GAs in WJ209, which is responsible for the dwarfism phenotype in sponge gourd.

## Supplementary Information

Supplementary file 1 (DOC 2795 kb)

Supplementary file 2 (XLSX 104 kb)

## Data Availability

The raw resequencing and transcriptome sequencing data are available from the NCBI under the project ID PRJNA606857.
