# Supplementary material for: A mutation in LacDWARF1 results in a GA-deficient dwarf phenotype in sponge gourd (Luffa acutangula)
Source: Theor Appl Genet. 2021 Aug 14;134(10):3443–57. doi: 10.1007/s00122-021-03938-4 (PMC8440308; doi:10.1007/s00122-021-03938-4)
Supplement: Supplementary file 1 — (DOC 2795 kb) [file 122_2021_3938_MOESM1_ESM.doc]

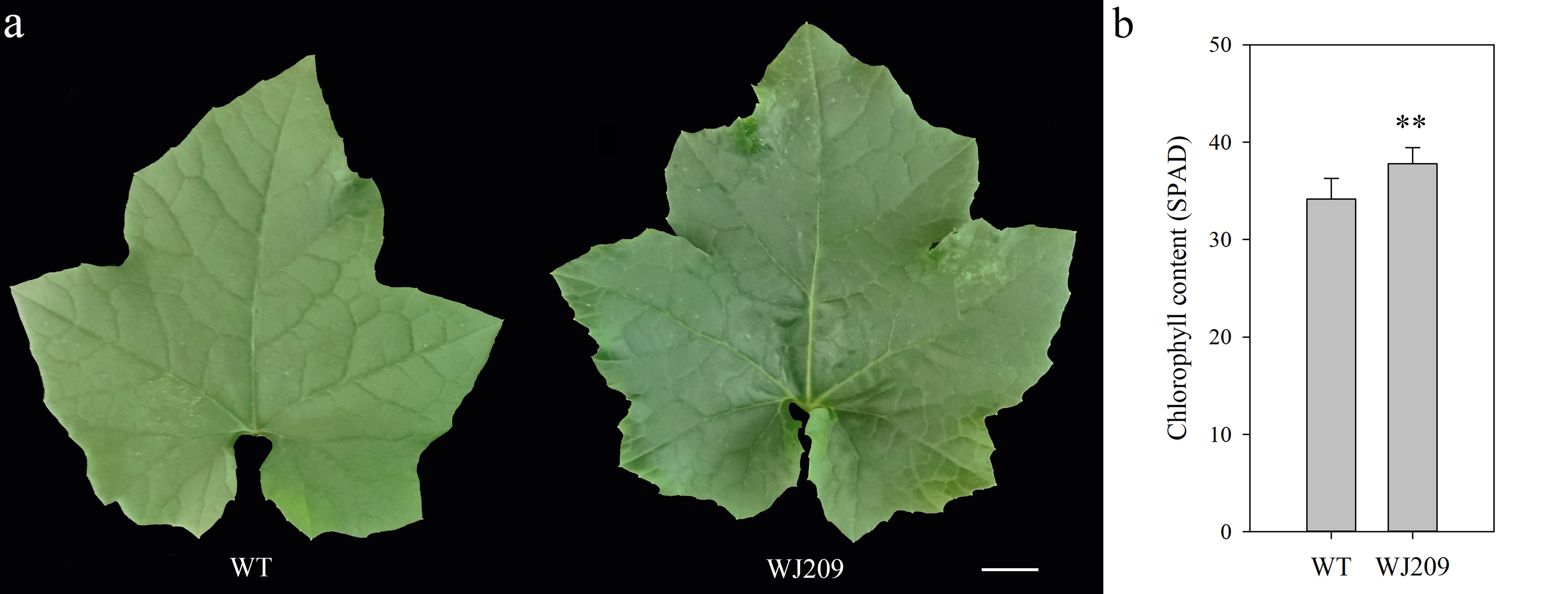


**Supplementary Fig. S1** Leaves of WT and WJ209. **a** Phenotypes of WT and WJ209. Bar, 1 cm. b SPAD of WT and WJ209. The asterisks indicate significant differences (Student’s t test): **p < 0.01, n ≥ 15. Error bars represent the mean ± SD.


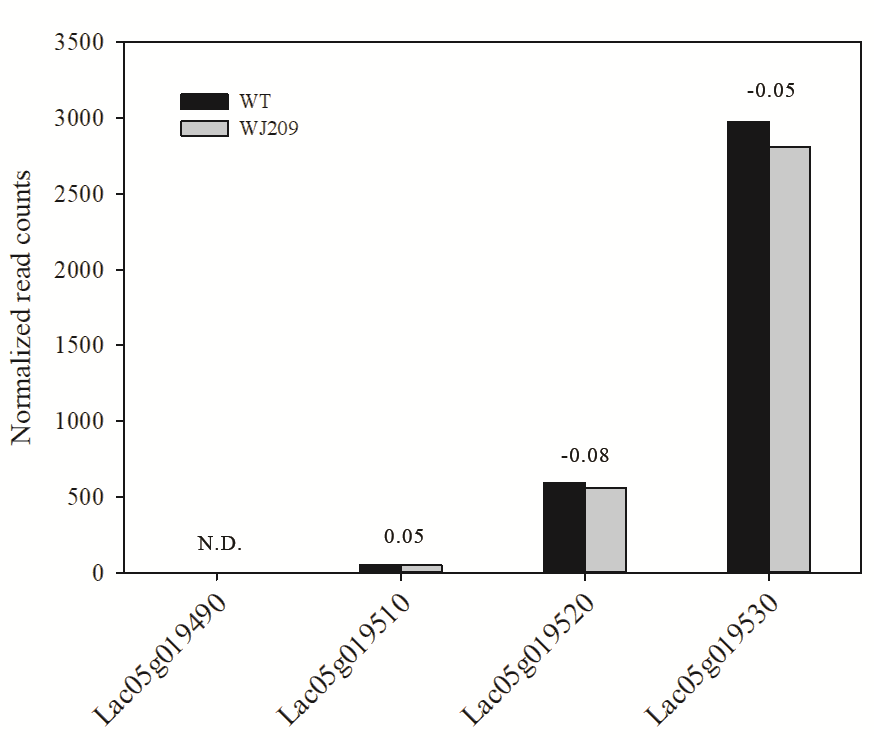


**Supplementary Fig. S2** Analysis of the expression of *Lac05g019490, Lac05g019510, Lac05g019520* and *Lac05g019530* between WT and WJ209


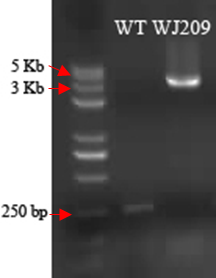


**Supplementary Fig. S3** Verify the insertion of *Lac05g019500* in WT and WJ209


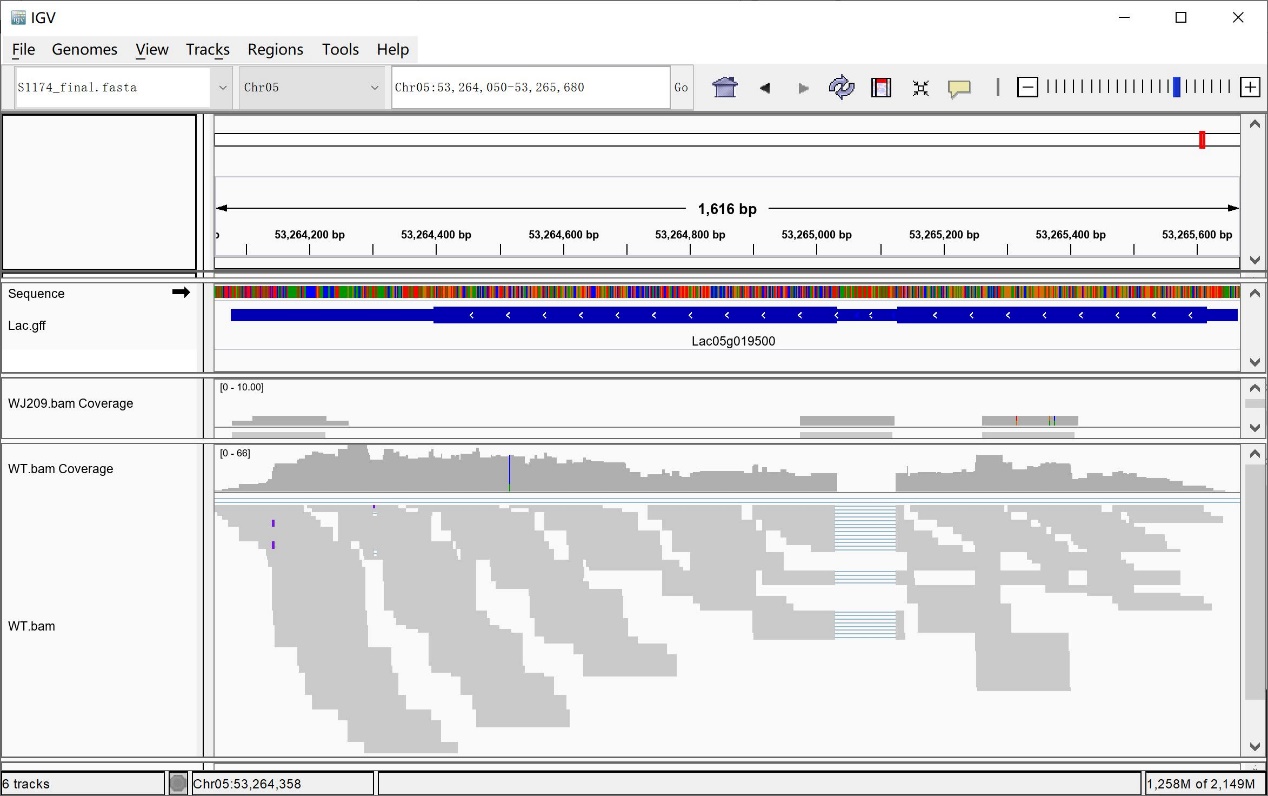


**Supplementary Fig. S4** Mapped RNA-Seq reads on *Lac05g019500*. Visualized by Integrative Genomics Viewer (IGV).


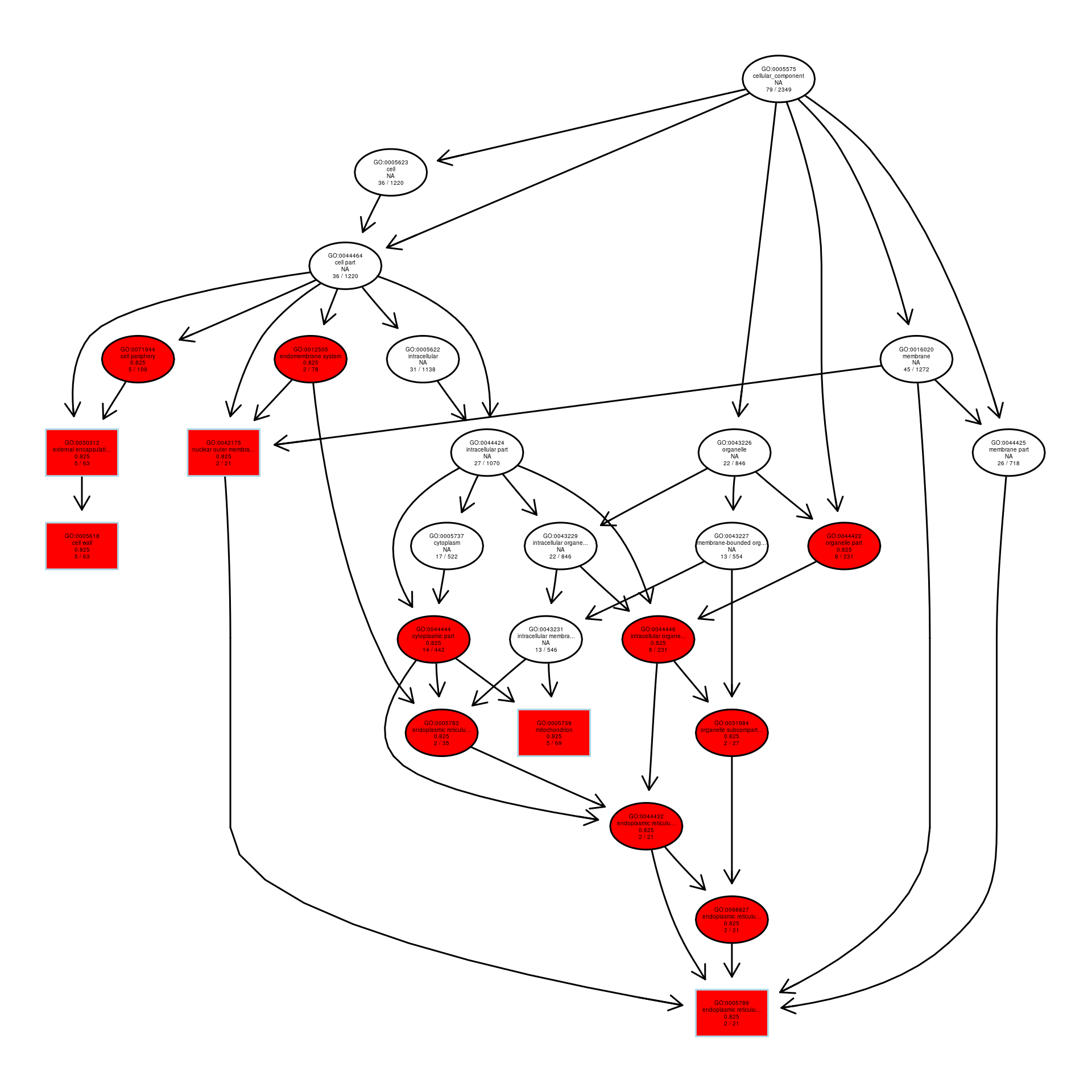


**Supplementary Fig. S5** DEGs enriched in cellular processes were significantly down-regulated


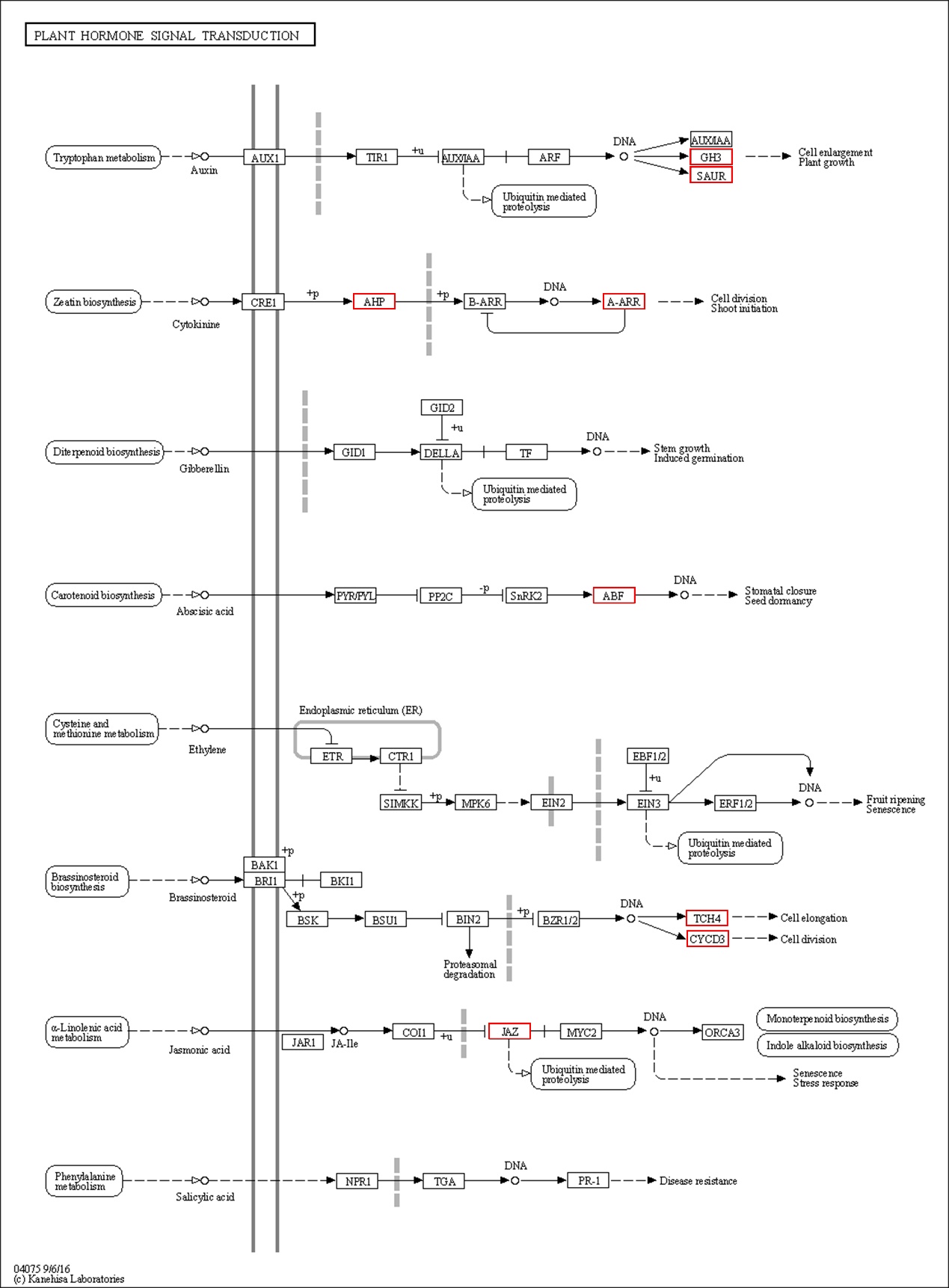


**Supplementary Fig. S6** Plant hormone signal transduction pathway. The red rectangles show changed genes.


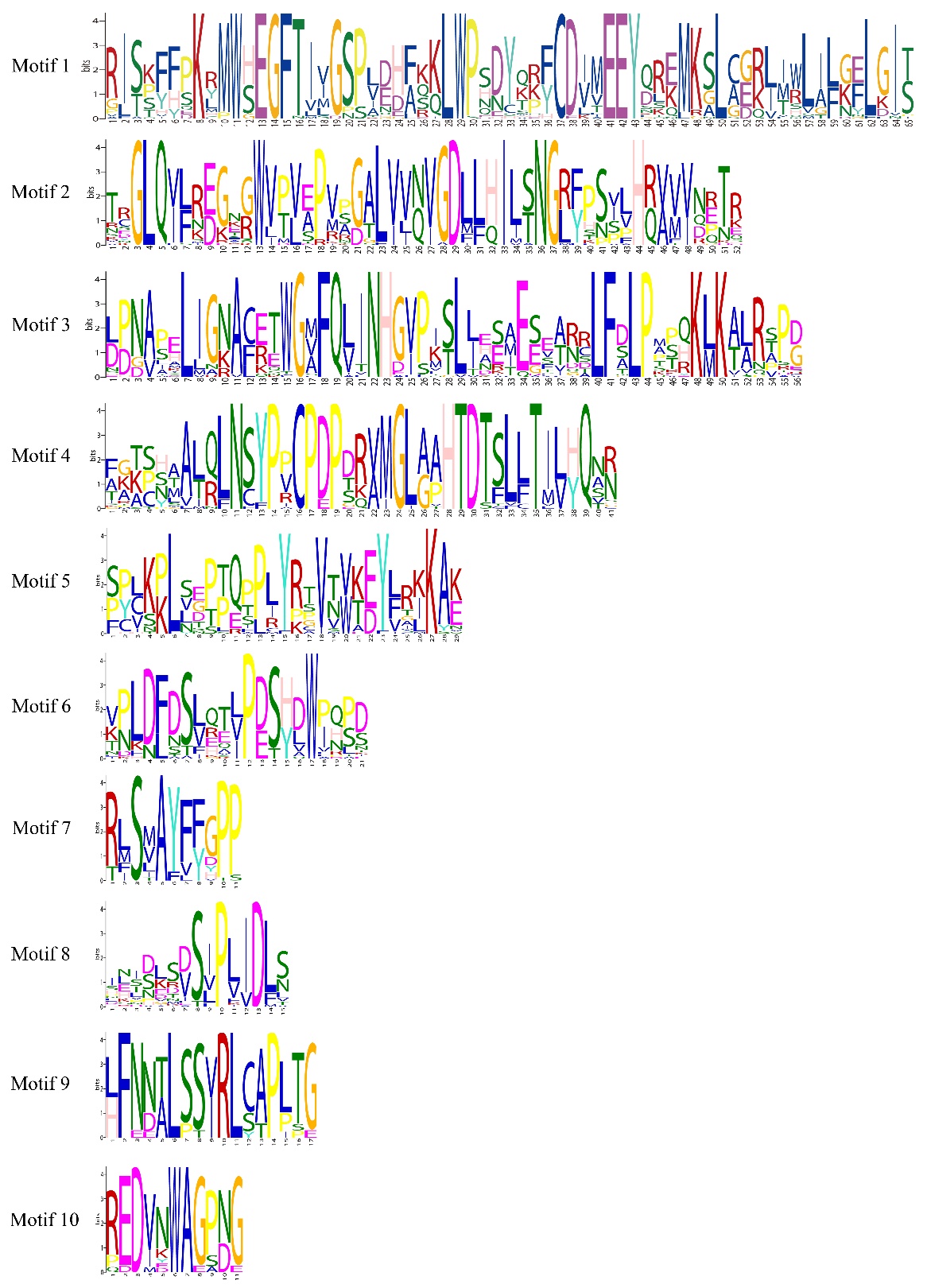


**Supplementary Fig. S7** Motifs sequences of GA3oxs in cucurbit species
